# Supplementary material for: Content-rich biological network constructed by mining PubMed abstracts
Source: BMC Bioinformatics. 2004 Oct 8;5:147. doi: 10.1186/1471-2105-5-147 (PMC528731; doi:10.1186/1471-2105-5-147)
Supplement: Additional File 2 — The original results of the above study (non-essential files are deleted to keep the file size under the limit set by BMC bioinformatics). [file 1471-2105-5-147-S2.bz2 › chilibotAdditionalFile2/dip20/748ID9811664E18130/html/EXBD_TONB.html]

 


 **EXBD** and **TONB** 
  
Found 54 abstracts in PubMed, retrieved 20.  
 

 What does Google say? 
 PDF only 
| .edu only 

---

**Interactive relationship** (e.g. stimulation, inhibition, etc)

**Neutral relationship**- The aim of this review is to describe an in vivo assay of the interactions taking place in the Tol Pal or  **TonB**  ExbB  **ExbD**  envelope complexes in the periplasm of Escherichia coli and between them and colicins or g3p protein of filamentous bacteriophages.  Ref: 12423784 Biochimie< MedlineTA>Biochimie,
- ExbB and  **ExbD**  proteins are part of the  **TonB**  dependent energy transduction system and are encoded by the exb operon in Escherichia coli.  Ref: 12193634 J Bacteriol, 2002
- Both ExbB and  **ExbD**  were also required for association of  **TonB**  with the cytoplasmic membrane.  Ref: 12193634 J Bacteriol, 2002
- Specifically, both ExbB and  **ExbD**  were found to be required for  **TonB**  to conformationally respond to proton motive force.  Ref: 12193634 J Bacteriol, 2002
- Cloning the mutation in the PhoA5 strain and sequencing the region adjacent showed that the mini Tn5 transposor interrupted the  **exbD**  gene, which forms part of the exbBD  **tonB**  operon.  Ref: 11514511 J Bacteriol, 2001

**Non-interactive relationship** (e.g. studied together, co-existance, homology, etc.)

- **TonB**  CysExbB  **ExbD**  D25N was still able to cross link with the FecA DALTV Cys derivatives in a  **tonB**  tolQ background, even though ExbD25N renders the  **TonB**  ExbBD complex nonfunctional V.  Ref: 12618451 J Bacteriol, 2003
- However, inducible levels of TX 100 resistance in gonococci had an absolute requirement for the  **TonB**  ExbB  **ExbD**  system, suggesting that such resistance in gonococci has an energy requirement above and beyond that required for constitutive pump activity.  Ref: 11796379 Antimicrob Agents Chemother, 2002
- **TonB** , the energy transducer, appears to go through a cycle during energy transduction, with the absence of both ExbB and  **ExbD**  creating blocks at two points i in the inability of  **TonB**  to respond to the cytoplasmic membrane proton motive force and ii in the conversion of  **TonB**  from a high affinity outer membrane association to a high affinity cytoplasmic membrane association.  Ref: 12193634 J Bacteriol, 2002
- Reverse transcription RT PCR, using RNA from organisms grown under iron replete and iron restricted conditions, revealed that  **tonB** , exbB,  **exbD** , tbpB and tbpA are transcribed as a single unit with expression being up regulated in response to iron restriction.  Ref: 12742718 Vet Microbiol, 2003
- PCR approaches allowed the amplification, cloning and sequencing of A. suis  **tonB** , exbB,  **exbD** , tbpB and tbpA homologues.  Ref: 12742718 Vet Microbiol, 2003
- In this study, we report the per cell numbers of  **TonB** , ExbB,  **ExbD**  and FepA for cells grown under iron replete and iron limited conditions.  Ref: 11967085 Mol Microbiol, 2002
- The Ton system consisting of the proteins  **TonB** , ExbB and  **ExbD**  is required for colicin and microcin import.  Ref: 12423780 Biochimie< MedlineTA>Biochimie, 2002
- Putative structural genes of the beta subunit of the histone like protein HU and of a new two component regulatory system were identified upstream from  **tonB**  and downstream from  **exbD** , respectively.  Ref: 10722583 Infect Immun, 2000
- In Gram negative bacteria, the  **TonB**  ExbB  **ExbD**  inner membrane multiprotein complex is required for active transport of diverse molecules through the outer membrane.  Ref: 11737642 Mol Microbiol, 2001
- The ratio of these proteins was calculated at one  **TonB**  two  **ExbD**  seven ExbB under all four growth conditions tested.  Ref: 11967085 Mol Microbiol, 2002
- They share homology with the ExbB,  **ExbD** , and  **TonB**  proteins, respectively.  Ref: 11418549 J Bacteriol, 2001
- The energy is transduced to FecA by the inner membrane complex,  **TonB** , ExbB, and  **ExbD** .  Ref: 12618451 J Bacteriol, 2003
- Following incubation on media containing hemoglobin, N. meningitidis IR3436  **tonB**  exbB  **exbD**  deletion mutant and N. gonorrhoeae PD3401  **tonB**  insertional mutant give rise to colonies which can grow with hemoglobin.  Ref: 10986265 J Bacteriol, 2000
- , Triton X 100 TX 100 did not require the  **TonB** , ExbB, or  **ExbD**  protein.  Ref: 11796379 Antimicrob Agents Chemother, 2002
- The reconstitution in Escherichia coli of the S. marcescens Has system demonstrated that haem uptake is dependent on the E. coli ExbB,  **ExbD**  and  **TonB**  proteins.  Ref: 11737642 Mol Microbiol, 2001
- A putative Fur binding sequence located upstream from  **tonB**  was detected in a Fur titration assay, indicating that the  **tonB**  exbB  **exbD**  operon may be Fur repressed in high iron growth conditions.  Ref: 10722583 Infect Immun, 2000
- The energy of the proton motive force is postulated to be transduced to the transport proteins by a protein complex that consists of the  **TonB** , ExbB, and  **ExbD**  proteins.  Ref: 11566987 J Bacteriol, 2001
- A complex of three membrane spanning proteins  **TonB** , ExbB and  **ExbD**  couples the chemiosmotic potential of the cytoplasmic membrane with siderophore uptake across the outer membrane.  Ref: 12409204 Biochim Biophys Acta, 2002
- Based on sequence similarities, the Bordetella pertussis  **tonB**  exbB  **exbD**  locus was identified on a cloned DNA fragment.  Ref: 10722583 Infect Immun, 2000
- In gram negative bacteria, high affinity iron uptake requires the  **TonB**  ExbB  **ExbD**  envelope complex to release iron chelates from their specific outer membrane receptors into the periplasm.  Ref: 10722583 Infect Immun, 2000
- ExbB and  **ExbD**  do not function independently in  **TonB**  dependent energy transduction.  Ref: 12193634 J Bacteriol, 2002
- Other biochemical evidence also shows that the proteins that provide energy for iron transport at the outer membrane, such as the  **TonB**  ExbB  **ExbD**  system, are structurally very similar across bacterial species.  Ref: 11895294 Curr Top Med Chem, 2001
- Inducible, but not constitutive, resistance of gonococci to hydrophobic agents due to the MtrC MtrD MtrE efflux pump requires  **TonB**  ExbB  **ExbD**  proteins.  Ref: 11796379 Antimicrob Agents Chemother, 2002
- Analysis of sequence alignments of TolQ and TolR homologues from numerous Gram negative bacterial genomes, together with analyses of the different tolQ tolR mutants, revealed that the TM domains of TolQ and TolR present structural and functional homologies not only to ExbB and  **ExbD**  of the  **TonB**  system but also with MotA and MotB of the flagellar motor.  Ref: 11722743 Mol Microbiol, 2001
- When tested on soybean host plants, hmuT hmuR and  **exbD**   **tonB**  mutants exhibited wild type symbiotic properties.  Ref: 11532144 Mol Microbiol, 2001
- Quantification of known components of the Escherichia coli  **TonB**  energy transduction system  **TonB** , ExbB,  **ExbD**  and FepA.  Ref: 11967085 Mol Microbiol, 2002
- In gram negative organisms, high affinity transport of iron substrates requires energy transduction to specific outer membrane receptors by the  **TonB**  ExbB  **ExbD**  complex.  Ref: 12562789 J Bacteriol, 2003
- Pasteurella multocida exbB,  **exbD**  and  **tonB**  genes are physically linked but independently transcribed.  Ref: 12044675 FEMS Microbiol Lett, 2002
- The exbB,  **exbD**  and  **tonB**  genes of the Pasteurella multocida animal pathogen have been cloned by complementation of an Escherichia coli  **tonB**  mutant.  Ref: 12044675 FEMS Microbiol Lett, 2002
- Crystal structures of two outer membrane transport proteins, FhuA and FepA, and biochemical data reveal strong long range conformational changes of the proteins upon binding of Fe 3 siderophore complexes and in response to energy transfer from the cytoplasmic membrane into the outer membrane via the  **TonB**  ExbB  **ExbD**  protein complex.  Ref: 11934617 Curr Opin Microbiol, 2002
